# The Prevalence of Dystonic Tremor and Tremor Associated with Dystonia in Patients with Cervical Dystonia

**DOI:** 10.1038/s41598-020-58363-2

**Published:** 2020-01-29

**Authors:** Lenka Hvizdošová, Martin Nevrlý, Pavel Otruba, Petr Hluštík, Petr Kaňovský, Jana Zapletalová

**Affiliations:** 1 Department of Neurology, Faculty of Medicine and Dentistry, Palacký University and University Hospital, I.P. Pavlova 185/6, 779 00 Olomouc, Czech Republic; 2 Department of Medical Biophysics, Faculty of Medicine and Dentistry, Palacký University Olomouc, Hněvotínská 976/3, 775 15 Olomouc, Czech Republic

**Keywords:** Epidemiology, Dystonia, Movement disorders

## Abstract

The link between dystonia and tremor has been known for decades, but the question of whether they are two separate illnesses or just different manifestations of one disease with the same pathophysiological background remains unanswered. We distinguish two types of tremor in dystonia: dystonic tremor (DT), which appears on the body part affected by dystonia, and tremor associated with dystonia (TAWD), which appears in locations where the dystonia does not occur. In this study, the frequency of occurrence of different forms of tremor was determined by clinical examination in a group of adult-onset isolated cervical dystonia (CD) patients treated with regular local injections of botulinum toxin A in our department. In total, 120 patients were included in the study, of which 70 (58.3%) had DT of the head. TAWD was, in all 14 cases (11.7%), observed on the upper limbs, in the form of static or intentional tremor. The aim of this study was to point out the presence of TAWD as one of the clinical signs of CD. DT occurred in more than half of the patients and appears to be a relatively common part of the clinical picture in patients with CD.

## Introduction

Dystonia was first described in 1911 by Oppenheim as a disorder in which involuntary muscle contractions lead to abnormal movements or postures^1^. This definition has since been revised many times as knowledge about the phenomenology, etiology, and pathophysiological mechanisms of dystonia have increased. The current definition describes dystonia as a movement disorder characterized by sustained or intermittent muscle contractions causing abnormal and often repetitive movements, postures, or both. Dystonic movements are typically patterned, twisting, and may be tremulous. Dystonia is often initiated or worsened by voluntary action and associated with overflow muscle activation^2^.

The current definition of dystonia includes tremor as one of the signs of a complex clinical presentation of dystonia. The occurrence of various types of tremor in dystonic movement disorders has been described in many studies. Its frequency ranges from 10 to 85%, depending on the studied population^3^. Two types of tremor are described in association with dystonia: DT, which appears in a body part affected by dystonia, and TAWD, which appears in locations where dystonia does not occur although the patient has dystonia elsewhere^4^.

DT can appear in any part of body affected by dystonia, but is most commonly present in CD as a head tremor or as a hand tremor in task-specific dystonia (e.g. writer’s cramp)^3^. Tremor can manifest during posture (static tremor) or voluntary movement (action tremor). Some patients have tremor at rest (rest tremor), which is often unilateral; in patients with bilateral rest tremor it usually prevails on one side^5^. DT most commonly manifests at or after dystonia onset^6^, but it can also precede the development of dystonia by many years, causing uncertainty about the diagnosis^7,8^. Unlike essential tremor (ET), DT can be suppressed by a sensory trick (geste antagoniste) and can disappear or be reduced in positions where dystonia tends to place the dystonic body part^9^. The frequency and amplitude of DT can be varied and irregular.

TAWD occurs in a body part that is not affected by dystonia. The most common example of this type of tremor is limb tremor in patients with CD. TAWD can manifest many years before, at the same time, or many years after dystonia onset^10,11^.

There have been many debates about the role of ET in patients with dystonia. Some studies favor the concept of coincidence of ET and CD^10,12–14^; some describe ‘ET plus dystonia’ as a subtype of ET^10,12^. On the other hand, some studies deny the coincidence or any association of dystonia with ET and support the concept of dystonia syndrome, which entails tremor as a possible sign of a complex clinical presentation of dystonia^10,11^.

In this study, we aimed to establish the prevalence of different types of tremor in patients with CD, treated with regular local injections of botulinum toxin A in our department, to summarize the current knowledge about these different types of tremor, and to point out the differences between TAWD and ET.

## Patients and Methods

Patients suffering from adult-onset isolated CD were recruited from the Movement Disorders Centre at the Department of Neurology, University Hospital, Olomouc, Czech Republic. The study was conducted in accordance with the Declaration of Helsinki 1964 (2013 revision) and it was approved by Ethics committee of University Hospital Olomouc. Patients with secondary CD or with segmental or multifocal dystonia were not included. All patients were over 18 years of age and gave informed consent before being included in the study. All patients were regularly treated with local injections of botulinum toxin A and the clinical assessment was performed at least three months after the last injection.

Subjects were clinically examined in detail by an experienced movement disorders specialist to exclude other forms of dystonia. During examination, the specialist focused on the presence of any involuntary movement in the face, upper limbs, lower limbs and also the trunk. The patients were asked in detail with the aim to explore any subjective symptoms of dystonic dyskinesia. The paraclinical and laboratory examinations, to exclude secondary form of dystonia, were always performed before the patient was recruited into study. In addition to the neurological examination, dystonia severity was assessed with the Toronto Western Spasmodic Torticollis Rating Scale (TWSTRS)^15,16^ and the Modified Tsui Scale for Cervical Dystonia^17,18^. The presence of tremor was assessed by a clinical examination of the patient in a resting state: head tremor when seated upright with head in a neutral position or when turning to either side; arm tremor when seated upright with hands placed comfortably on thighs (rest tremor), outstretched in pronation (postural tremor), or during a finger-to-nose maneuver (kinetic tremor). We also looked for tremor in other body parts during the clinical examination and while taking patient’s history (i.e. voice tremor during sustained vowels, lower limb tremor during heel-to-shin testing), but none of the patients showed any signs of tremor elsewhere.

All statistical analyses were performed using IBM SPSS Statistics software, version 22 (IBM Corporation, USA). Continuous variables were described as median and range. The Kruskal-Wallis test was used to reveal the relationship between the head and/or upper limb tremor and quantitative parameters. The Fisher’s exact test was used to evaluate the relationship between tremor and qualitative parameters. Normal distribution was verified by the Shapiro-Wilk test. A significance level less than 0.05 was considered statistically significant (P < 0.05).

## Results

In total, this study involved 120 patients, of which 25 were men (20.8%) and 95 were women (79.2%). The mean patient age was 59.3 years, ranging from 29 to 90. The mean treatment length was 8.4 years. The patients’ clinical characteristics are detailed in Tables 1 and 2.

**Table 1:** Characteristics of patient group.

	Mean	SD	Minimum	Maximum	Median
Age	59.3	13.2	29.0	90.0	60.0
Treatment length (in years)	8.4	5.4	0.2	26.0	8.0
TSUI	5.2	3.1	1.0	18.0	5.0
TWSTRS	14.2	5.0	2.0	28.0	14.0

SD – standard deviation, TSUI - Modified Tsui Scale for Cervical Dystonia, TWSTRS - Toronto Western Spasmodic Torticollis Rating Scale.

**Table 2:** Characteristics of dystonia type in patient group.

	Torticollis		Laterocollis		Antecollis	Retrocollis
	right	left	right	left		
Present	42 (35.0%)	59 (49.2%)	31 (25.8%)	26 (21.7%)	5 (4.2%)	14 (11.7%)

**Figure 1:**
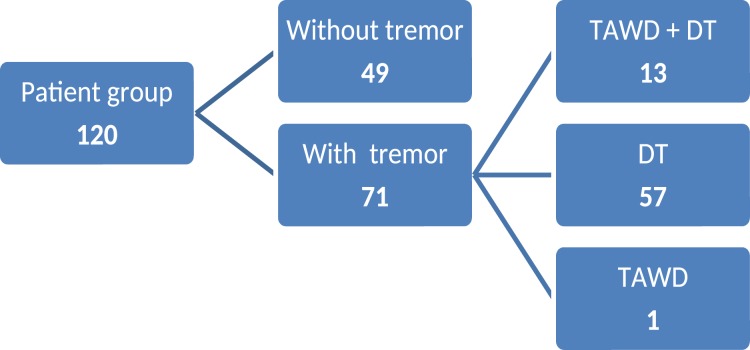
Tree diagram showing prevalence of different types of tremor in patient group.

Various types of tremor were present in a total of 71 patients (Fig. 1). In most cases, tremor was present as DT of the head (70 patients, 58.3%). Fourteen patients (11.7%) had TAWD on the upper limbs, 13 of which also had DT of the head; only one patient had only tremor of the upper limbs. In terms of sex, both types of tremor occurred much more frequently in female patients. The female-to-male ratio in DT was 11:3; in TAWD, it was 13:1. TAWD was not present in the resting state. All 14 patients with TAWD had upper limb tremor in a static posture; 7 of them (5.85%) also had TAWD during movement, notably when approaching the target during the finger-to-nose maneuver.

Dystonia severity was assessed with TWSTRS and the Modified Tsui Scale for Cervical Dystonia. The mean TWSTRS score was 14.2, ranging from 2 to 28. When using the Modified Tsui Scale for Cervical Dystonia, the mean score was 5.2 points, with a minimal score of 1 and a maximum score of 18.

For further comparison, we divided the patients into three subgroups: patients with TAWD and DT, with DT and without tremor (Table 3). Subgroup of patients with TAWD was not included in the comparison because of small group sample.

**Table 3:** Characteristics of patient subgroups.

		DT + TAWD		DT		Without tremor		Fisher’s exact test p
		Count	%	Count	%	Count	%	
Treatment length	> = 13 years	3	23.1%	15	26.3%	19	38.8%	0.323
	<13 years	10	76.9%	42	73.7%	30	61.2%	
Sex	men	1	7.7%	14	24.6%	10	20.4%	0.450
	women	12	92.3%	43	75.4%	39	79.6%	

DT – dystonic tremor, TAWD – tremor associated with dystonia.

No statistical significance was observed in relation to dystonia severity or length of treatment and the occurrence of tremor (Table 4). In relation to age, the p-value of the Kruskal-Wallis test was <0.05, but there was no significant difference between the subgroups when compared with the Dunn’s post hoc test.

**Table 4:** Comparison between patient subgroups depending on age, treatment length, and dystonia severity.

		DT + TAWD	DT	Without tremor	Kruskal-Wallis test p
Age	Median	67.0	61.0	58.0	0.0489
	Minimum	43	37	29	
	Maximum	83	90	82	
Treatment length (in years)	Median	6.0	8.0	10.0	0.408
	Minimum	2	1	0	
	Maximum	14	26	23	
TSUI	Median	4.0	5.0	4.0	0.424
	Minimum	2	1	1	
	Maximum	12	18	15	
TWSTRS	Median	13.0	15.0	14.0	0.438
	Minimum	2	2	5	
	Maximum	19	24	28	

TSUI - Modified Tsui Scale for Cervical Dystonia, TWSTRS - Toronto Western Spasmodic Torticollis Rating Scale, p – probability value, DT – dystonic tremor, TAWD – tremor associated with dystonia.

We divided patients according to various types of dystonic head and neck movements: right or left torticollis and laterocollis, retrocollis, and anterocollis. We found no statistical significance in the occurrence of hand tremor according to type of dystonic movement.

## Discussion

In this study, tremor was a common accompanying feature of isolated adult-onset CD, occurring in more than half of the patients. In previously published studies, the occurrence of tremor ranged from 10 to 85%, depending on the studied population^3^. This wide range of occurrence of tremor in dystonia could be attributed to various factors, including small patient samples, evaluation of only one or, on the other hand, of various types of dystonia, using only medical chart reviews instead of clinical examinations to evaluate tremor, or from including patients with acquired dystonia. Genetic variations may also impact the phenotypic spectrum of dystonia and tremor.

Previous studies observed a higher preponderance of DT and TAWD in female rather than male populations^5,6,19^. Our study confirms this, even though the patient group was mainly composed of female patients, with a female-to-male ratio of 19:5. This sex preponderance has been repeatedly observed and studied, but no clear explanation has been described^20^.

According to the current definition of dystonia, dystonic movements may be tremulous, and this tremor may precede the development of dystonia by many years^7,8^. This raises the question of whether patients with various types of tremor who do not yet display evident signs of dystonia are correctly diagnosed and appropriately treated, especially if invasive therapy, such as deep brain stimulation, is being considered. Among tremulous disorders, the most over-diagnosed one seems to be ET. Diagnosis of ET is based purely on clinical criteria. ET is defined as bilateral, largely symmetrical, postural or kinetic tremor affecting hands and forearms that is visible and persistent^10,21^. Various studies reviewed patient cohorts diagnosed as pure ET, and found associated features of dystonia in different body parts^22,23^, or features of other neurological disorders, such as Parkinson’s disease^24,25^. According to Jain *et al*.^25^, about one in three patients with tremor was misdiagnosed as having ET, with the most often false diagnoses being Parkinson’s disease and dystonia. Louis *et al*. indicate that misdiagnosis of ET occurs in 30 to 50% of cases, suggesting that it could be the most commonly misdiagnosed movement disorder^25,26^. Misleading factors in the diagnosis of ET might include that CD patients with DT may also show alcohol responsiveness or have autosomal dominant familial history^5,27^. Overall, there seems to be a high overlap between dystonia and tremor and one can easily be missed if other is subtle.

Other signs might suggest CD instead of ET during clinical examination. For example, DT, unlike ET, may respond to sensory tricks (geste antagoniste) in which touching a specific body part leads to the reduction of tremor or dystonic posture. Agnew *et al*.^28^ studied the prevalence of tremor in CD and ET patients in a supine position and while seated upright. Their results indicate that head tremor in patients with ET is a postural tremor that is reduced or completely disappears when a patient lies down, whereas in patients with CD, head tremor more often persists.

Another often over-diagnosed clinical entity seems to be an isolated head tremor as a part of a phenotypic spectrum of ET. Louis *et al*.^26^ studied a population of 583 ET patients and found none with isolated head tremor with a complete absence of arm tremor, and only 2.7% of patients with head tremor and only mild arm tremor.

Why tremulous disorders are so often misdiagnosed remains unknown, but it indicates the need for more precise, revised criteria and definitions of these disorders. The exact pathophysiological background of dystonic and tremulous disorders remains unknown and requires further studies.

We acknowledge that our study might have some limitations. It is not a population-based study, but a cross-sectional study, focused only on CD patients. We focused only on patients with focal CD; we did not include patients who, apart from CD, showed dystonic movements elsewhere. The results of prevalence studies for tremor in patients with more widespread forms of dystonia might differ. We did not consider the duration of symptoms before the beginning of treatment and we also did not include data of tremor versus dystonia onset because of the possible high inaccuracy of retrospectively collected data. Untreated comparison group of CD patients would also be very important to determine if treatment affects the presence of associated tremor and also if severity of untreated dystonia is in correlation with tremor presence or its type.

## Conclusion

Data from this study estimates the prevalence of different types of tremor in patients with CD. DT seems to be a frequent accompanying feature in CD patients (58.3%); TAWD seems to be much less common (11.7%). We point out differences between tremor in dystonia and in ET, probably the most commonly misdiagnosed condition in this field. In light of these observations, we encourage physicians to also look for signs of dystonia in non-tremulous body parts during clinical examination of patients with tremor.
